# Profiling Anthocyanins in Thai Purple Yams (*Dioscorea alata* L.)

**DOI:** 10.1155/2020/1594291

**Published:** 2020-07-09

**Authors:** Subin Srivichai, Parichat Hongsprabhas

**Affiliations:** Department of Food Science and Technology, Faculty of Agro-Industry, Kasetsart University, Bangkok 10900, Thailand

## Abstract

Two accessions of Thai purple yam (*Dioscorea alata*) were investigated for their chemical constituents during tuber development when the vines were 3 to 8 months old. Yam tubers contained total phenolic compounds ranging between 100 and 385 mg gallic acid equivalent (GAE), flavonoids 60–160 mg catechin equivalent (CE), monomeric anthocyanin of 10–90 mg cyanidin-3-glucoside equivalent (CGE), 70 g starch, and 25–30 g amylose in 100 g yam tuber on a dried weight basis, depending on the accession and age of yam vine. LCMS-IT-TOF mass spectrometry revealed that anthocyanins in both accessions from 8-month-old vines had cyanidin or peonidin nucleus. Their glycosides were nonacylated, monoacylated, or diacylated with sinapic or ferulic acid. The major yam anthocyanins found in both accessions were alatanin C (cyanidin 3-(6-sinapoyl gentiobioside). This study revealed the insights on chemical components during tuber development and characteristics of alatanins for future selection and cultivation of purple yam tubers.

## 1. Introduction

*Dioscorea* spp. is one of the most economically important crops serving as a staple food for millions of people in tropical and subtropical countries [1]. It is mainly grown for human consumption in Africa, South America, and South Pacific [2]. In South-East Asia, the well-known species cultivated in the Philippines are *Dioscorea alata* and *Dioscorea esculenta* [3]. Yam bioactive compounds include steroidal saponins called diosgenin and dioscin, storage protein dioscorin, and anthocyanins [4–9]. The bioactivities and health benefits of yam extracts include antioxidant capacity, immunomodulatory activity, antihypertensive activity, antimicrobial activity, estrogenic effect, and antitumor effect [4–9].

Although *Dioscorea* comprises more than 600 species, only 15–20 species are edible, and some are used as medicinal food [10]. The Chinese yams, namely, *D. opposita* Thunb., *D. alata* L., *D. fordii* Prain et Burkill, and *D. persimilis* Prain et Burkill of 25 germplasms are a good source of nutrient and bioactive compounds allantoin and dioscorin for medicinal use [9]. However, the diversity of nutrients and bioactive compounds quantitatively varied within the same species [2, 9].

Some yam species have attractive purplish colours, i.e., *D. alata* or *ube* yam from the Philippines, *raja-ala* from Sri Lanka [11], and *D. trifida* L. from South America [12]. Anthocyanins in *D. alata* are cyanidin–based such as cyanidin 3-*O*-gentiobioside; alatanin 1 [13]; alatanin 2 [13]; alatanins A, B, and C [14]; and alatanins D, E, F, and G [15]. The purple pigment in *D. trifida* L., however, was mainly peonidin glycoside [12]. The aglycone anthocyanin may be species-dependent.

Given recent trends towards the natural food colour, the food industry is much interested in coloured crops as the source of natural pigments. Unlike the well-known South-East Asian purple yam *ube* from the Philippines, many species of *Dioscorea* in Thailand are under-utilised and considered endangered since they encounter the threat of biodiversity loss. Some species have been used domestically due to its unique colour, texture, and flavour compared to those of sweet potato and cassava tubers. Nonetheless, most edible and medicinal yam tubers consumed in Thailand are wild tubers collected from the forest and not from cultivation.

We have previously reported the utilisation of yam flour and starch from a selected germplasm collection from the Department of Agriculture, Ministry of Agriculture and Co-operatives, Thailand [16, 17], as well as post harvest changes of polyphenolic compounds and antioxidant capacities during storage of yam tuber [18]. Anthocyanins are known as unstable molecules that are highly susceptible to degradation induced by pH, temperature, anthocyanin concentration, oxygen, light, enzymes, ascorbic acid, sugars, and sulfite [19]. The stability of anthocyanins in yam flour, therefore, needs to be considered for their further uses.

Although yam purple pigments exhibit potential health benefits as mentioned earlier, the study on the types of yam anthocyanins from South-East Asia is limited and merits further investigation. The degradation of yam anthocyanins may occur during growth, after harvesting, during starch extraction, anthocyanin extraction, processing of yam-based foods, and storage of coloured yam products. This study is aimed at investigating the changes in chemical compounds during tuber development of purple yam and at identifying the anthocyanins of the mature tubers. The insights from this study could yield the information for yam breeding, cultivation, and utilisation programme in the future.

## 2. Materials and Methods

### 2.1. Materials

Germplasms of accession Khon Kaen Field Crop Research Center (acc. KKFCRC) and accession BKJ from Kasetsart University was sprouted during the rainy season in July 2016 at the plantation site in Phra Nakhon Si Ayutthaya, central plain region, Thailand, by local farmers. Fresh tubers from each vine (*n* = 3 vines) were harvested when the vines were 3, 4, 5, 6, 7, and 8 months old before the dormancy period started in April 2017. The shape, weight, and size of whole yam tubers were recorded after washing. The cleaned tubers were stored at room temperature (27°C) for one day unless noted otherwise.

### 2.2. Effects of Accession and Vine Age on Chemical Compounds in Yam Tuber

#### 2.2.1. Sample Preparation

Tubers harvested at different vine ages were washed at the plantation site, cut into 10 × 10 × 10 mm cubes and kept in liquid N_2_ before transportation to the Department of Food Science and Technology, Faculty of Agro-Industry, Kasetsart University, Bangkok, Thailand, within two h after harvest. The frozen samples were stored at -18°C for one day before extraction for the analyses.

Yam water extract was prepared by mixing five grams of the frozen sample with liquid N_2_ and 25 mL of water, centrifuged at 3500 g at 4°C for 10 min under refrigeration at 10°C (Thermo Scientific, Sorvall Biofuge Stratos, Milford, Massachusetts, USA). The supernatant was filtered through Whatman No. 1 filter paper and kept in amber bottles at -18°C before analyses.

#### 2.2.2. Total Phenolic Content

Total phenolic content of yam water extract was determined by mixing 0.1 mL of an extract with 0.1 mL Folin-Ciocalteu's phenol reagent, 1.0 mL 7% Na_2_CO_3_, and 1 mL of distilled water. The reaction was allowed to proceed at room temperature for 90 min [20]. Absorbance at 760 nm was measured by a microplate reader (TECAN Infinite® 200 PRO, Grödig, Austria) and expressed as mg gallic acid equivalent (GAE) in 100 g yam (d.b.).

#### 2.2.3. Flavonoid

Flavonoid content of yam water extract was determined spectrophotometrically [21]. Briefly, 0.25 mL of the water extract prepared was mixed with 1.25 mL of distilled water and 0.075 mL of 5% NaNO_2_ solution and allowed to react for 5 min. Then, the 0.15 mL of 10% aluminium chloride was added, and the reaction proceeded for 6 min before the addition of 0.5 mL of 1 mol L^−1^ NaOH. Distilled water was added to bring the final volume of the mixture to 3 mL. The absorbance of the mixture was measured at 510 nm against a prepared blank using a Tecan microplate reader. The flavonoid content was determined using a catechin standard curve and expressed as mg of catechin equivalent (CE) in 100 g yam (d.b.).

#### 2.2.4. Monomeric Anthocyanin

Monomeric anthocyanin content of yam water extract was determined by the pH-differential method [22] using two buffer systems: 0.025 mol L^−1^ potassium chloride buffer, pH 1.0 and 0.4 mol L^−1^ sodium acetate buffer, pH 4.5. Yam water extract (0.20 mL) mixed with either 0.80 mL of 0.025 mol L^−1^ potassium chloride buffer pH 1.0 or a 1.00 mL of 0.4 mol L^−1^ sodium acetate buffer pH 4.5. The reactions proceeded for 15 min, and the absorbance was read at 510 and 700 nm using a Tecan microplate reader. The total monomeric anthocyanin content, expressed as mg cyanidin-3-glucoside equivalent (CGE) in 100 g yam (d.b.), was calculated based on the difference in absorbance and molar absorptivity of cyanidin 3-glucoside (*Ɛ*) of 26,900.

#### 2.2.5. Polyphenol Oxidase Activity

Five grams of frozen yam was mixed with liquid N_2_ and buffer (0.2 mol L^−1^ sodium phosphate buffer at pH 7.0 with 0.25% (*v*/*v*), Triton X-100 and 5% (*w*/*v*) polyvinylpyrrolidone (PVPP)). The suspensions were centrifuged at 17257 g for 30 min at 4°C (Thermo Scientific; Sorvall Biofuge Stratos; MA, USA). The supernatant was collected and stored at -18°C. Enzyme activity was determined using the method described by [23]. The reaction mixture containing 3.5 mL of 0.20 mol L^−1^ phosphate buffer (pH 6.8), 1 mL of 0.05 mol L^−1^ catechol, and 0.5 mL of enzyme solution was monitored as absorbance at 410 nm using the Tecan microplate reader. The rate of the reaction was calculated from the initial linear slope of the activity curves. The enzyme unit was defined as the changes in absorbance per min.

#### 2.2.6. Soluble Protein Content

The protein content in the supernatant prepared as described in section 2.2.5 was quantified using the Bradford method [24]. The 0.1 mL sample was mixed with 1 mL protein reagent (0.01% (*w*/*v*) Coomassie Brilliant Blue G-250, 4.7% (*w*/*v*) EtOH, and 8.5% (*w*/*v*) phosphoric acid). The absorbance at 595 nm was measured after 2 min using the Tecan microplate reader.

#### 2.2.7. Starch and Amylose Contents

The yam tuber cubes were blended in a Waring blender and dried at 40°C for 24 h using Memmert ULM 400 hot air oven (Memmert GmbH+ Co. KG, Schwabach, Germany). The dried samples were ground and passed through a 100-mesh sieve, packed in a sealed aluminium foil pouch, and stored at -18°C. The starch and amylose contents were analysed enzymatically using the total starch (AA/AMG) assay kit and the amylose/amylopectin test kit, respectively (Megazyme, Wicklow, Ireland).

### 2.3. Identification of Yam Anthocyanins in EtOH Extracts from Different Accessions

Freshly harvested yam tubers of the KKFCRC and BKJ accessions of the eight-month-old vines were peeled, chopped in a Waring blender, and dried at 40°C for 24 h using the Memmert ULM 400 hot air oven. The dried samples were ground and passed through a 100-mesh sieve, packed in a sealed aluminium foil, and stored at -18°C. Five grams of sample were mixed with 25 mL of acidified 50% EtOH of pH 2.4 for 30 min at 25°C and centrifuged at 3000 g for 10 min in an Ohaus Frontier 5706 centrifuge four times (Parsippany, New Jersey, USA). The supernatant was evaporated using a CentriVap benchtop centrifugal vacuum concentrator (Kansas, USA) at 40°C. The remaining solid was dissolved in a 50% EtOH (*v*/*v*), adjusted the final volume to 2 mL, and kept at -18°C in an amber bottle before analysis.

The sample solution (2 mL) was passed through a C-18 Sep-Pak cartridge (VertiPak™ C18-LP) previously activated with MeOH, followed by 3 mL of 0.1% HCl (*v*/*v*) in deionised water. The anthocyanins and polyphenolics were adsorbed onto the Sep-Pak column while sugars, acids, and other water-soluble compounds were removed by washing the column with 5 mL 0.01% HCl in water. Anthocyanins were recovered with 2 mL of 50% EtOH (*v*/*v*) containing 0.1% HCl (*v*/*v*). This solution was stored at -20°C. Samples were filtered through a 0.45 *μ*m filter before analysis.

Twenty microliters of samples was injected into the LCMS-IT-TOF (SHIMADZU, Tokyo, Japan) coupled with Prevail™ C18 column (5 *μ*m, 150 × 4.6 mm). The LC gradient followed a linear gradient elution program, with 0.5% (v/v) aqueous trifluoroacetic acid (TFA) (mobile phase A(and 80% (*v*/*v*) acetonitrile in MeOH (mobile phase B) at a flow rate of 0.05 mL min^−1^ started from 0% phase B and increased from 0 to 5% for 10 min and from 5 to 15% from 10 to 20 min; maintained at 15% from 20 to 25 min; increased from 15 to 20% from 25 to 30 min; increased from 20 to 50% from 30 to 50 min; maintained at 50% from 50 to 60 min; and decreased from 50 to 0% from 60 to 61 min. The wavelength of the UV–visible detector is at 520 nm.

Mass spectrometry parameters (MS) were as follows: capillary voltage, 4.5 kV; interface temperature, 200°C; heat block temperature, 200°C; gas flow (N_2_), 1.5 L min^−1^. The instrument was operated in positive ion mode scanning from *m*/*z* 100 to 2500, collision gas (argon) pressure 50%, and collision energy 50%.

### 2.4. Effect of Accession and Flour Preparation on the Colour of Yam Flour

Mature yam tubers from both accessions were harvested, cleaned, and separated into two groups. The flour from fresh tuber was prepared by chopping the edible portion in a Waring blender and dried at 40°C for 24 h using the Memmert ULM 400 hot air oven (Memmert GmbH+ Co., Schwabach, Germany). The dried samples were ground and passed through a 100-mesh sieve and stored at -18°C. The other portion of yam tuber was sliced to 2 cm without peeling and steamed at 100°C for one h, cooled down to 25°C by soaking in cold water, peeled and chopped in a Waring blender, and dried at 40°C for 24 h using a hot air oven. The dried samples were ground and passed through a 100-mesh sieve and stored at -18°C.

### 2.5. Statistical Analysis

The data were analysed by using analysis of variance (ANOVA) at a 95% significance level. When significant differences were found among mean values, post hoc analysis was determined by Duncan's multiple range tests. All statistical analyses were performed using SPSS Software Version 12 (SPSS Inc., USA).

## 3. Results and Discussion

### 3.1. Effects of Accession and Vine Age on Chemical Compounds in Yam Tuber

The growth of yam vine and yam tuber can be divided into 3 phases. The first phase is the sprouting period, defined as the time that the mother tuber germinated [25]. The second phase is the regrowth period or the time required for the new tuber to form and the seedling of the new vine. The new vine uses nutrients from the mother tuber and absorbs soil nutrients through the roots during this period. The third phase is the tuberisation period. It is the period that the new tuber starts to develop and synthesise starch. This phase usually starts after sprouting for 2 to 3 months.

**Figure 1:**
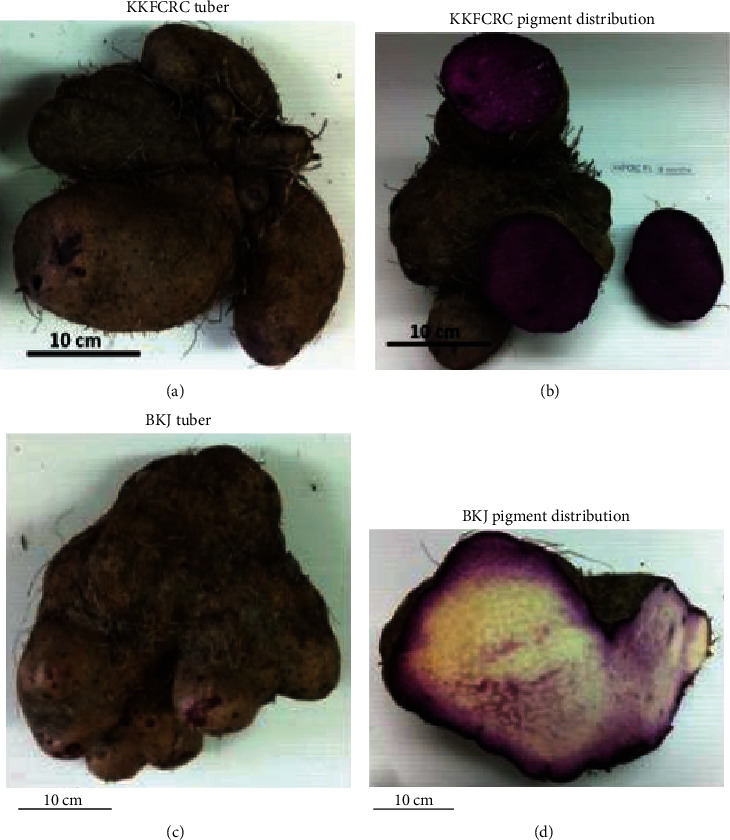
Appearance of tuber and purple pigment distribution in *Dioscorea alata* flesh from 8-month-old vine.

Thai purple yam of both accessions began sprouting in July (rainy season) and was harvested in April of the following year when the leaves started withering. Harvested tubers had variable shapes with the majority being cylindrical. The numbers of mature tubers varied from 1 to 5 tubers in each vine, as shown in Figure 1. The purple pigment in the KKFCRC tuber was evenly distributed in the edible portion throughout their age, while the pigment in the BKJ tuber was concentrated closer to the peel, particularly in the older vine when the starch content was high.

**Figure 2:**
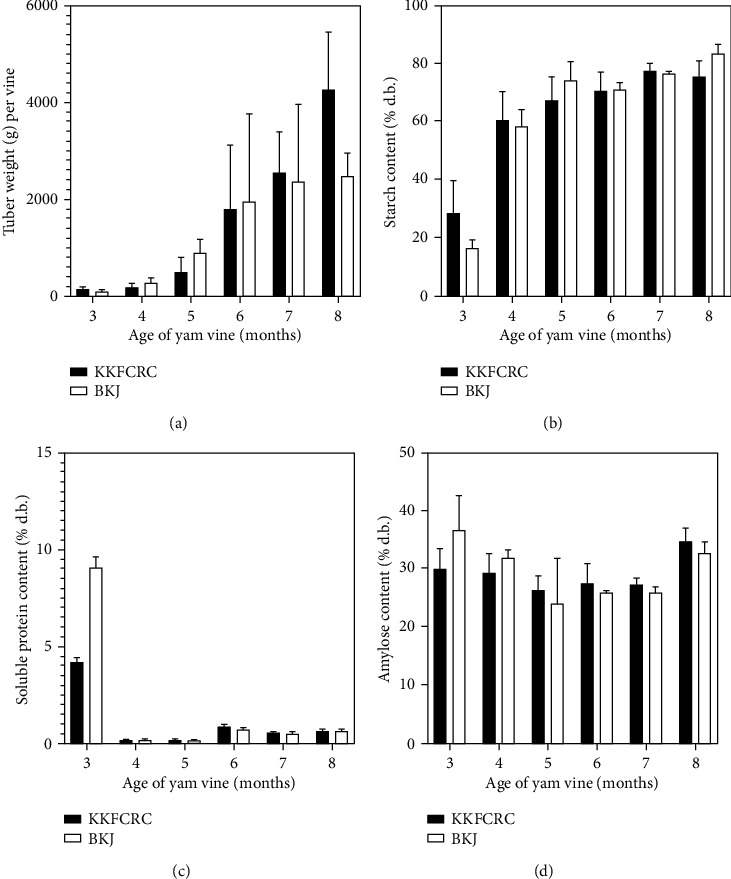
Effect of vine age and accession (KKFCRC vs BKJ) on yam tuber qualities: (a) tuber weight, (b) starch, (c) protein content, and (d) amylose content in yam tuber. Bars represent standard deviation.

Figure 2(a) shows that the eight-month-old vine generated tubers having an average weight of 2 to 4 kg, depending on the accession (*P* < 0.05). The starch content in the KKFCRC and the BKJ tubers drastically increased after the vine was four months old, and it reached at least 75% (d.b.) at the time of harvest or eight months of age (Figure 2(b)). Nonetheless, the amylose content around 25–35% (Figure 2(d)) could be considered high for tuber starches [11]. The soluble protein content in tubers, however, showed a reduction during the development of tuber. Tubers from the 6 to 8-month-old vine contained around 1% soluble proteins (Figure 2(c)).

**Figure 3:**
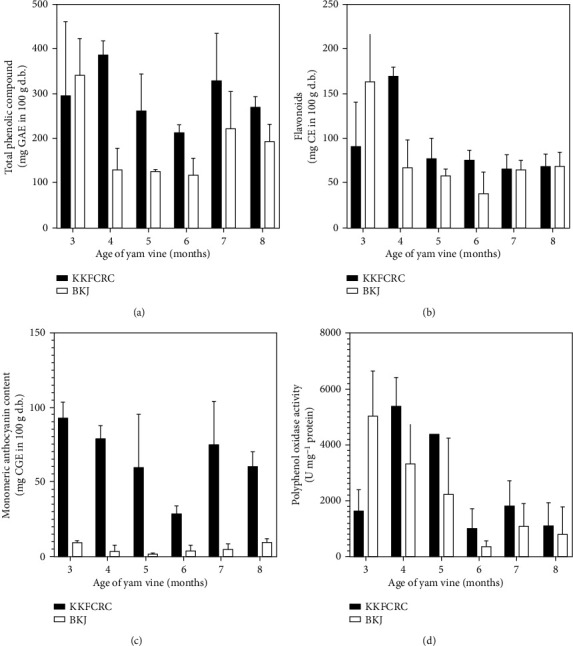
Effect of vine age and accession (KKFCRC vs BKJ) on chemical characteristics of the edible portions: (a) total phenolic compound, (b) flavonoid, (c) monomeric anthocyanin, and (d) polyphenol oxidase activity. Bars represent standard deviation.

A two-way ANOVA revealed that the vine age did not significantly affect the total phenolic compounds and the anthocyanin (*P* ≥ 0.05), but the age influenced flavonoid content and the polyphenol oxidase (PPO) activity (*P* < 0.05; Figure 3). The total phenolic compounds (Figure 3(a)) and anthocyanin contents (Figure 3(c)), however, depended on the yam accession. The KKFCRC yam contained much higher anthocyanin than did the BKJ yam (*P* < 0.05). At the time of harvest, the KKFCRC yam tuber had monomeric anthocyanin around 60.6 mg CGE in 100 g (d.b.), which was almost ten times higher than that of the anthocyanin found in the BKJ tuber.

PPOs in plants are responsible for the prevention of pathogens and insects that are harmful to them [26].  Figure 3(d) shows that the PPO activity was high in the tuber harvested from the vine before five months. However, the PPO activity in both KKFCRC and BKJ tubers declined before harvesting.

### 3.2. Identification of Yam Anthocyanins in EtOH Extracts from Different Accessions

**Figure 4:**
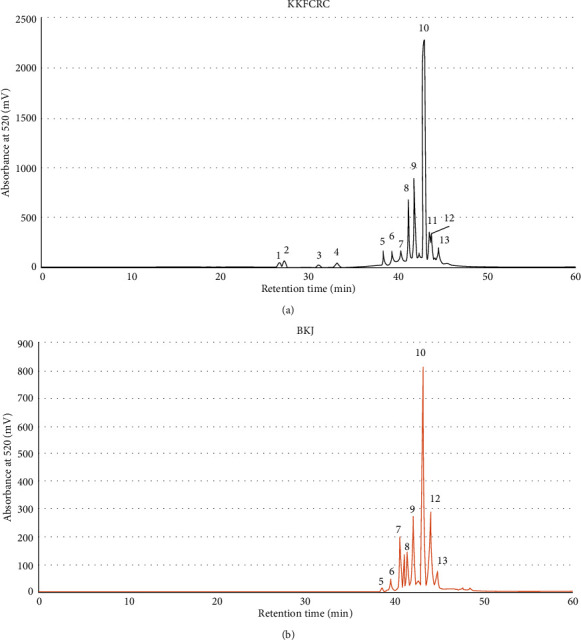
LC profile of the EtOH extracts of the (a) KKFCRC and (b) BKJ tuber accessions at 520 nm. Peak numbers correspond to KKFCRC anthocyanins in Table 1.

The chromatograms shown in Figure 4 indicated different profiles of anthocyanins in EtOH extracts of the KKFCRC and BKJ tubers. Out of 13 peaks detected in the KKFCRC extract, 11 peaks were identified as anthocyanins. The extract from the BKJ tuber, however, had a lower content of anthocyanin than the KKFCRC extract (Figure 4(b)).

The data on retention time, molecular ion precursor ([M]^+^), and the primary fragment ions (*m*/*z*) of the KKFCRC yam are presented in Table 1. Mass spectral characteristics of the extract indicated that peaks 1, 4, and 7 had the same precursor [M]^+^ at 611 but different retention times. From their fragmentation patterns, they were most likely cyanidin glycoside isomers, which are commonly found in *Dioscorea* species [2, 12, 15].

Peaks 1 and 7 shared the same MS spectra but different retention times. Peak 1 showed the precursor [M]^+^611 gave one MS^2^ fragment ion at *m*/*z* 287 (cyanidin). They were tentatively identified as cyanidin 3-diglucoside. The MS data of peak 4 showed two MS^2^ fragment ions at *m*/*z* 449 ([M]^+^–162 mass unit of glucose) and at *m*/*z* 287. This peak 4 was identified as cyanidin 3, 5-diglucoside. A notable difference in the retention times among peaks 1, 4, and 7 (i.e., 26.78, 33.23, and 40.40 min) was observed. Peak 1 is likely to be an isomer with a higher polarity than those of peaks 4 and 7. Peak 7 (retention time 40.4 min) was found in both the KKFCRC and BKJ tubers.

Peak 2, which was found only in the KKFCRC extract, had a molecular ion precursor [M]^+^773. The mass spectral data suggested that this anthocyanin was cyanidin glycosylated with three glucose molecules, i.e., cyanidin-3-diglucoside, 5-glucoside.

Peaks 6 and 8 showed the same molecular ion precursor [M]^+^ at 979 and similar mass spectral data but different retention times and were found only in the KKFCRC extract. Peak 6 shows three fragment ions: MS^2^*m*/*z* 817 (i.e., [M]^+^–162 mass unit of glucose), *m*/*z* 449, and *m*/*z* 287 (cyanidin). The molecular ion precursor in peak 8 fragmented into MS^2^*m*/*z* 449 [M–tri-glucose–sinapic acid]^+^ and 287 (cyanidin). The MS data suggested that anthocyanins observed as peak 6 and peak 8 were composed of a cyanidin nucleus, three glucose units, and one (*E*)-sinapic acid unit. The notable difference in retention times (39.41 and 41.26 min) was observed, and peak 6 was tentatively identified as alatanin E or cyanidin-3-*O*-(sinapoyl)-diglucoside-5-*O*-glucoside as recently reported [15] as acylated anthocyanins in purple yam from the Philippines. Peak 8 reported in this study, however, could be alatanin E isomer. It was less polar than the peak 6 alatanin E as the notable difference in retention time, and the fragmentation pattern was evident. Alatanin E and the newly discovered isomer found in the KKFCRC yam were absent in the BKJ yam (Table 1).

Peak 9 in the KKFCRC extract showed molecular ion precursor [M]^+^ at 1347 and three fragment ions MS^2^*m*/*z* at 979 ([M]^+^–glucose–sinapic acid), *m*/*z* 817, and *m*/*z* 449, suggesting that this anthocyanin had a cyanidin, four glucose units, and two (*E*)-sinapic acid unit. Peak 9 was tentatively identified as alatanin B, as previously reported [14].

The main yam anthocyanins in EtOH extracts were identified as alatanin C (peak 10). It was present in both KKFCRC and BKJ tubers (Figure 4). Peak 10 had a molecular ion precursor at [M]^+^817 and MS^2^ fragment ions *m*/*z* 449 and *m*/*z* 287. The MS data indicated that this anthocyanin contained a cyanidin, two glucose units, and one (*E*)-sinapic acid unit. Peaks 10 were tentatively identified as alatanin C, as previously reported by [14].

The presence of both alatanin C and alatanin B was previously reported as the major anthocyanin in *D. alata* grown in the Philippines [14, 15]. From MS fragmentation patterns, alatanin C was previously identified as 3-*O*-(6-*O*-(6-*O*-(*E*)-sinapoyl-*β*-D-glucopyranosyl)-*β*-D-glucopyranosyl)cyanidin and alatanin B as 3-*O*-(6-*O*-(6-*O*-(*E*)-sinapoyl-*β*-D-glucopyranosyl)-*β*-D-glucopyranosyl)-7-*O*-(6-*O*-(*E*)-sinapoyl-*β*-D-glucopyranosyl)-2′-*O*-(*β*-D-glucopyranosyl)cyanidin [14].

Peak 11 in the KKFCRC extract had a molecular ion precursor [M]^+^ at 787 and one fragment ion MS^2^ m/z 287, suggesting that it was composed of a cyanidin nucleus, two glucose units, and one (*E*)-ferulic acid unit. Peak 11 was found only in the KKFCRC extract and tentatively identified as alatanin G isomer. It had a different fragment pattern from alatanin G previously reported in the yam from the Philippines [15].

Peak 12, which was found in both KKFCRC and BKJ yam tubers, had a molecular ion precursor at [M]^+^1185 and two fragment ions MS^2^*m*/*z* 817 and *m*/*z* 449. The MS data suggested that this anthocyanin had one cyanidin nucleus, three glucose units, and two (*E*)-sinapic acid units. Peak 12 was tentatively identified as alatanin D isomer because it had a different fragment pattern from the alatanin D identified previously [15].

Peak 13 had a molecular precursor at [M]^+^831 and one fragment ion MS^2^*m*/*z* 301, suggesting that this anthocyanin had a peonidin unit, two glucose units, and one (*E*)-sinapic acid unit. Peak 13 was tentatively identified as alatanin F isomer since it had a different fragment pattern from the alatanin F reported earlier [15]. This alatanin F isomer was found in the KKFCRC and the BKJ yam tubers.

This study revealed the changes in chemical components during the development of two tuber yam accessions. Compared to a major root crop such as cassava, which is commonly grown in South-East Asia, purple yam could be a potential source of anthocyanins that are not affected by the vine age.

**Figure 5:**
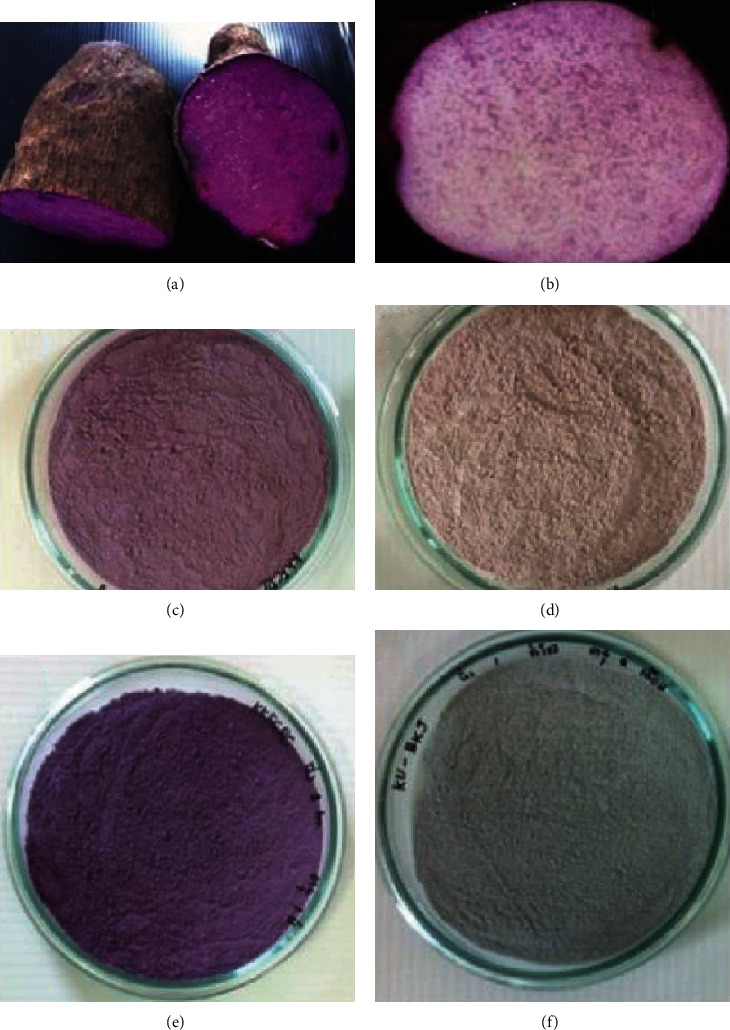
Effect of steaming tuber before milling on the colour of yam flour from KKFCRC (a, c, e) and BKJ (b, d, f) tubers: (a, b) flesh tuber colour, (c, d) flour prepared directly from tubers, and (e, f) flour prepared from steamed tubers.

The effect of tuber steaming on purple colour stability of yam flours prepared from the KKFCRC and BKJ accessions was studied. The KKFCRC flour made from the fresh tuber lost the intense purple colour, as shown in Figure 5. The BKJ flour, which had lower anthocyanins, was brownish pink (Figure 5(d)) compared to the light purplish tuber of the KKFCRC flour (Figure 5(c)). This loss of colour may be due to the PPO activity and the different anthocyanin profiles in the KKFCRC and BKJ yam tubers. Steaming the tubers before flour milling could help reduce enzymatic browning reactions and retain the purple colour of the yam flour, especially the colour of the KKFCRC flour (Figure 5(e)). The BKJ flour prepared from the steamed tuber was light grey to blue, as shown in Figure 5(f). Nonetheless, the residual PPO in the yam tuber may need to be inactivated to preserve its intense purple colour.

## 4. Conclusions

The majority of anthocyanins found that two Thai purple yam-cultivated accessions had a cyanidin nucleus with alatanin C (cyanidin 3-(6-sinapoyl gentiobioside)) as the main anthocyanin in both accessions. The type and content of minor alatanins (cyanidin or peonidin monoacylated and diacylated with sinapic or ferulic acid) were accession dependent and could influence the intensity of the purple colour in yam flour.

## Figures and Tables

**Table 1:** Mass spectrometric data and tentative identification of anthocyanins in the KKFCRC yam EtOH extracts.

Peak no.	Retention time (*t*_R_, min)	Area (%)	Molecular ion precursor [M]^+^ (*m*/*z*).	MS^2^ fragment ion (*m*/*z*)	Tentative identification	Molecular formula	Remarks
1	26.78	1.25	611	611[M]^+^→287	Cyanidin-3-diglucoside	C_27_H_31_O_16_	
2	27.29	1.92	773	—	Cyanidin-3-diglucoside, 5-glucoside	C_33_H_41_O_21_	
3	31.17	1.09	Unknown	Unknown	Unknown	Unknown	
4	33.23	1.81	611	611[M]^+^→449, 287	Cyanidin-3,5-diglucoside	C_27_H_31_O_16_	
5	38.44	4.46	Unknown	Unknown	Unknown	Unknown	
6	39.41	2.79	979	979[M]^+^→817, 449, 287	Alatanin E	C_44_H_51_O_25_	Identified by [15]
7	40.40	4.59	611	611[M]^+^→287	Cyanidin-3-diglucoside	C_27_H_31_O_16_	
8	41.26	11.13	979	979[M]^+^→449, 287	Alatanin E isomer	C_44_H_51_O_25_	Different fragment pattern from alatanin E identified by [15]
9	41.90	16.13	1347	1347[M]^+^→979, 817, 449	Alatanin B	C_61_H_71_O_34_	Identified by [14]
10	42.97	38.49	817	817[M]^+^→449, 287	Alatanin C	C_38_H_41_O_20_	Identified by [14]
11	43.60	5.52	787	787[M]^+^→287	Alatanin G isomer	C_37_H_39_O_19_	Different fragment pattern from alatanin G identified by [15]
12	43.84	5.73	1185	1185[M]^+^→817, 449	Alatanin D isomer	C_55_H_61_O_29_	Different fragment pattern from alatanin D identified by [15]
13	44.66	5.08	831	831[M]^+^→301	Alatanin F isomer	C_39_H_43_O_20_	Different fragment pattern from alatanin F identified by [15]

## Data Availability

The data used in this study are available from the authors upon request.
